# Floral Anatomy, Sporogenesis, and Gametogenesis in the Rubber Dandelion (*Taraxacum kok-saghyz*): Implications for Breeding and Crop Development

**DOI:** 10.3390/plants15071036

**Published:** 2026-03-27

**Authors:** Carolina Schuchovski, Tea Meulia, Bruno Francisco Sant’Anna-Santos, Elaine Lopes Pereira Nunes, Jonathan Fresnedo-Ramírez

**Affiliations:** 1 Department of Biological Sciences, Kent State University, 800 E. Summit St., Kent, OH 44242, USA; cschucho@kent.edu; 2 Molecular and Cellular Imaging Center, The Ohio State University, 1680 Madison Avenue, Wooster, OH 44691, USA; meulia.1@osu.edu; 3 Laboratório de Anatomia e Biomecânica Vegetal, Departamento de Botânica, Setor de Ciências Biológicas, Universidade Federal do Paraná, Avenida Coronel Francisco H. dos Santos, 100, Centro Politécnico, Jardim das Américas, C.P. 19031, Curitiba 81531-980, PR, Brazil; brunofrancisco@ufpr.br; 4 National Coalition of Independent Scholars, 125 Putney Road, Brattleboro, VT 05301, USA; elaine.nunes@ncis.org; 5 Department of Horticulture and Crop Science, The Ohio State University, 1680 Madison Avenue, Wooster, OH 44691, USA

**Keywords:** Asteraceae, flower anatomy, gametogenesis, neo-domestication, rubber dandelion, sporogenesis, tribe Cichorieae

## Abstract

*Taraxacum kok-saghyz* (TK), the rubber dandelion, is an emerging crop offering potential for sustainable natural rubber production independent of tropical climates. Successful domestication of TK requires a mechanistic understanding of its reproductive biology, yet floral anatomy, sporogenesis, and gametogenesis remain poorly characterized. We hypothesized that TK’s reproductive development follows the general patterns of sexually reproducing diploid *Taraxacum* species and other Asteraceae, distinguishable from the irregular meiosis reported in apomictic taxa. Here, using light and scanning electron microscopy across multiple developmental stages, we describe the floral and inflorescence anatomy, as well as sporogenesis and gametogenesis in TK. Anther development in TK predominantly follows the simultaneous microsporogenesis pattern, typical of eudicots, producing regular tetrahedral tetrads. Notably, we also observed occasional successive-type events resulting in dyads and tetragonal tetrads, indicating a previously unreported developmental variation within the species, culminating in mature tricellular pollen. We detail key reproductive structures, including anther wall layers, ovary mesophyll differentiation, and the presence of a micropylar obturator. The meiotic behavior and gametophyte development observed in TK are consistent with those of diploid, sexually reproducing *Taraxacum* species and other members of the Asteraceae, in contrast to the irregular meiosis reported in *Taraxacum* apomictic taxa. These newly described morphoanatomical details on reproductive aspects will inform breeding strategies and advance our understanding of pollination, fertilization, and seed development in TK.

## 1. Introduction

Natural rubber is a ubiquitous material across modern industry, with demand increasing year after year. However, the global supply chain rests on one species, *Hevea brasiliensis* Müll.Arg, leaving the economy vulnerable to conditions in the few tropical regions capable of natural rubber production. The introduction of alternative crops would add much-needed resilience to disease and habitat loss [1,2]. One promising candidate is the rubber dandelion (*Taraxacum kok-saghyz* Rodin—hereafter TK), an annual crop that produces high-quality natural rubber, has a short life cycle, produces a substantial amount of biomass, and does not require a tropical climate [3,4].

Despite recent advances such as improved rubber yield, plant vigor, and genomic tools, traits such as self-incompatibility and high heterozygosity have impeded domestication in TK [4,5,6]. Multiple strategies have been applied to address these challenges, including CRISPR/Cas9 genome editing [7], the development of molecular markers [8,9], and transcriptomic studies [10,11]. Despite this progress, the cellular and developmental mechanisms governing TK’s reproductive biology—from anther wall formation through gametophyte maturation—have not been documented in systematic histological detail.

The genus *Taraxacum* belongs to the family Asteraceae, subfamily Cichorioideae, tribe Cichorieae, and sub-tribe Crepidinae [12]. *Taraxacum* includes around 60 sections and 2800 species [13] with different ploidy levels, chromosome numbers, and sexual or apomictic reproduction strategies [13,14,15]. Within this taxon, reproductive strategy usually aligns with ploidy level: diploid species typically present sexual reproduction, while polyploid species are generally apomictic [16]. In keeping with this pattern, the diploid *Taraxacum kok-saghyz*, with 2n = 16 chromosomes, presents sexual reproduction and is predominantly self-incompatible [14,17]. While several studies have explored floral and reproductive traits across *Taraxacum* species—both sexual and apomictic [15,16,18,19,20,21,22,23]—detailed anatomical and developmental descriptions in TK are scarce. Existing work has primarily focused on early embryology [17] and general floral development [24], leaving significant gaps in our knowledge of floral anatomy and both male and female sporogenesis and gametogenesis. Understanding reproductive development in TK is essential for effective breeding programs and long-term crop improvement, as it directly influences fertility, seed production, and the ability to select for desirable traits [6,25].

We hypothesize that TK’s floral anatomy and reproductive development follow general patterns seen in other sexually reproducing diploid *Taraxacum* species and related Asteraceae. Specifically, we predict that TK will exhibit: (i) simultaneous microsporogenesis producing tetrahedral tetrads, (ii) unitegmic and tenuinucellate ovules with a chalazal functional megaspore, and (iii) tricellular, tricolporate pollen—all features consistent with sexual diploid *Taraxacum* and inconsistent with the irregular meiosis documented in apomictic polyploid taxa. Deviation from these predictions would suggest novel reproductive pathways potentially linked to TK’s self-incompatibility or its unique domestication bottleneck.

This study therefore aims to: (1) provide a comprehensive histological description of TK inflorescence and floral anatomy; (2) characterize male sporogenesis and microgametogenesis, including anther wall development and pollen maturation; (3) characterize female sporogenesis and megagametogenesis; and (4) compare these features with sexual and apomictic *Taraxacum* species and other Asteraceae to evaluate conserved versus species-specific traits relevant to TK breeding.

## 2. Results

### 2.1. Inflorescence Anatomy and Development

**Figure 1:**
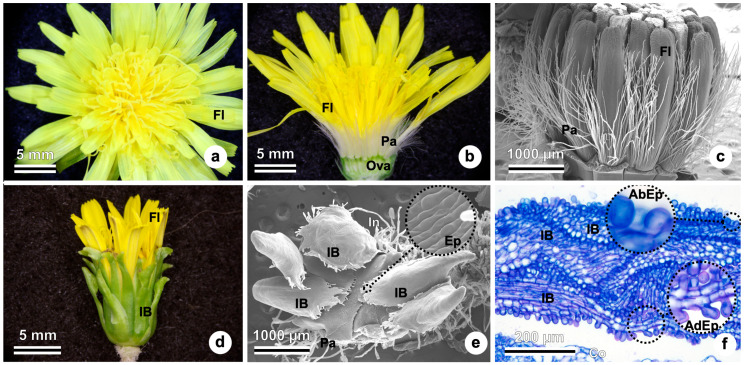
Inflorescence in *Taraxacum kok-saghyz*. (**a**) Top view of an inflorescence at anthesis with ligulate flowers observed in stereomicroscopy. (**b**) Longitudinal view of an inflorescence, with flowers showing pappus and ovary observed in stereomicroscopy. (**c**) Developing inflorescence showing flowers with visible pappus in scanning electron microscopy. Involucral bracts removed. (**d**) Developing inflorescence with opened involucral bracts surrounding the flowers observed in stereomicroscopy. (**e**) Developing inflorescence with closed involucral bracts surrounding the flowers in scanning electron microscopy. (**f**) Anatomy of involucral bracts in a developing inflorescence, showing the abaxial and adaxial papillate epidermis in light microscopy. AbEp, abaxial epidermis; AdEp, adaxial epidermis; Ep, epidermis; Fl, flowers; IB, involucral bracts; Ova, ovary; Pa, pappus.

The anthetic inflorescence in TK is a solitary capitulum formed by many yellow ligulate florets with white pappus (Figure 1a,b). Figure 1c shows details of the developing inflorescence with visible pappus elements surrounding the florets. Involucral bracts can be observed in Figure 1e,f. The opening inflorescence is seen in Figure 1d, showing protecting involucral bracts, with details in Figure 1e,f. The enclosing involucral bracts show abaxial and adaxial epidermis (Figure 1f).

**Figure 2:**
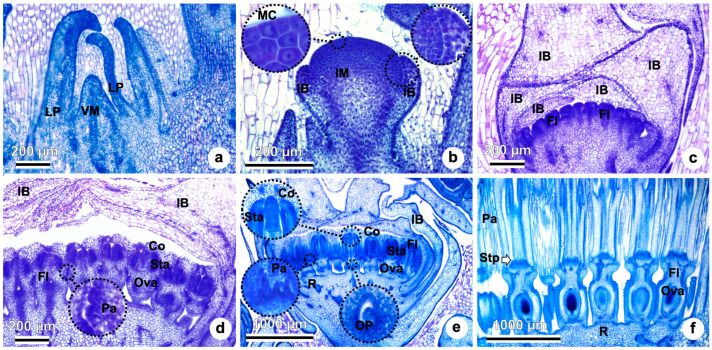
Inflorescence anatomy and development of *Taraxacum kok-saghyz*. (**a**) Vegetative meristem surrounded by leaf primordia. (**b**) Transition to inflorescence meristem, with involucral bracts on its flanks. Meristematic cells on detail. (**c**) Inflorescence meristem with many flower primordia formed centripetally, covered by involucral bracts. (**d**) Developing pappus (in detail), corolla, androecium, and gynoecium in each flower. (**e**) Developing inflorescence meristem with pappus, corolla, androecium, and gynoecium; pappus elements, ovule primordia, and corolla in details (**f**) Developed stage of the inflorescence with stylopodia (gynoecial nectaries) visible. Abbreviations: Co, corolla; Fl, flowers; IB, involucral bracts; IM, inflorescence meristem; LP, leaf primordia; MC, meristematic cells; OP, ovule primordia; Ova, ovary; Pa, pappus; R, receptacle; Sta, stamens; Stp, stylopodium; VM, vegetative meristem.

The development of the vegetative meristems (VM) and inflorescence meristems (IM) start in the center of the basal rosette. The VM is elongated and surrounded by many leaf primordia (Figure 2a). Next, the VM transitions into an IM (Figure 2b). The young IM is flatter than the VM and bears involucral bracts on its flanks (Figure 2b). As the inflorescence develops, many floral primordia are formed centripetally and enclosed by the involucral bracts (Figure 2c). From each of these flower primordia, corolla, androecium, pappus, and gynoecium develop (Figure 2d,e). Pappus elements start forming above the ovary (Figure 2d,e). The single ovule primordium is already seen (in detail) within the recently formed ovary (Figure 2e). A more developed inflorescence stage is seen in Figure 2f with stylopodia (gynoecial nectaries) visible in the apex of the ovary.

### 2.2. Flower Anatomy

**Figure 3:**
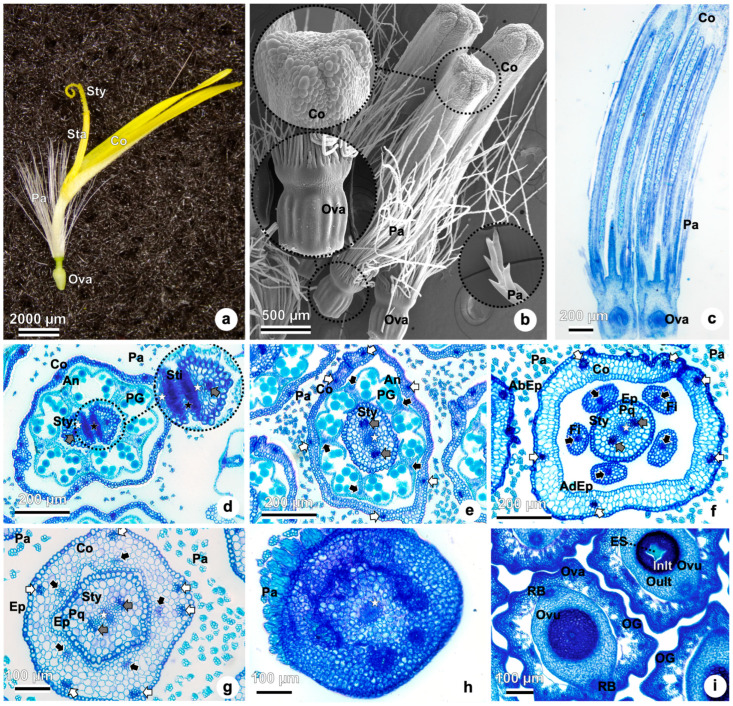
Flower anatomy of *Taraxacum kok-saghyz*. (**a**) Stereoscopy (**b**) Scanning electron microscopy (**c**) Longitudinal view at light microscopy. (**d**–**i**) Transversal views in light microscopy of flowers at different levels, starting from the distal point at the anthers level and going to the proximal level where ovaries are located. (**a**–**c**) Flowers showing pappus, corolla, androecium, and gynoecium. (**d**) Corolla enclosing the androecium. Five anthers with introrse dehiscence releasing pollen grains. Pappus elements surrounding the corolla. In the center of the flower, the gynoecium, the style is visible, with vascular bundles (gray arrow) and the pollen tube transmitting tissue (white star). The stigmatic tissue shows many papillate cells (black star). (**e**) Anthers are connate. Vascular bundles from the corolla (6 white arrows), from the stamens (5 black arrows), and the style (2 gray arrows). Style with the pollen tube transmitting tissue in the center (white star). (**f**) Corolla shows a thicker layer of parenchymatic cells with six vascular bundles (white arrow). The adaxial epidermis shows more elongated cells. Filaments are separated from the corolla. Style with epidermis, parenchyma, two vascular bundles (gray arrow), and pollen tube transmitting tissue (white star). (**g**) Corolla at the proximal level has more layers of parenchymatic cells. Five vascular bundles from the filaments (black arrow). At this stage, filaments are adnate to the corolla. Pappus elements surrounding the corolla. Style in the center. (**h**) Pappus elements above the ovary, pollen tube transmitting tissue in the center (white star). (**i**) At the level of the inferior ovary with many outgrowths. Ovule showing a unitegmic integument with distinct inner and outer zones and the embryo sac. Rapheal bundle observed in the ovary. Abbreviations: AbEp, abaxial epidermis; AdEp, adaxial epidermis; An, anthers; Co, corolla; Ep, epidermis; ES, embryo-sac; Fi, filament; InIt, inner zone of the integument; OG, outgrowths; OuIt, outer zone of the integument; Ova, ovary; Ovu, ovule; Pa, pappus; PG, pollen grains; Pq, parenchyma; RB, rapheal bundle; Sta, stamens; Sti, stigma; Sty, style.

The flowers in the capitulum are bisexual, showing the corolla, androecium, pappus, and gynoecium in different stages of development (Figure 3a–c). Three flowers were detached, allowing the corolla, inferior ovary, and pappus to be observed in detail (Figure 3b). The corolla oblong epidermal cells are visible at the distal region of the flower.

Floral anatomy was examined in both longitudinal (Figure 3c) and transverse sections at various levels from the anther region to the ovary within flowers at the same developmental stage (Figure 3d–i).

In the transverse section of the floret (Figure 3d), a thin corolla encloses five anthers, which exhibit introrse dehiscence, releasing pollen grains toward the center of the flower. Surrounding the corolla, pappus bristles are visible at the periphery. The vascular bundles of the style are evident, along with the stigmatic tissue and the pollen tube transmitting tissue.

Stamens are connate by their anthers, forming a tube around the gynoecium (Figure 3d,e). At this level, the corolla is composed of several layers of parenchymatic cells. The adaxial epidermis of the corolla exhibits more elongated cells than the abaxial side. The filaments of the five stamens are distinct from the corolla, and the style is characterized by two vascular bundles and a central transmitting tissue (Figure 3f). In more proximal sections (Figure 3g), the corolla consists of five petals and six main vascular bundles. Additional parenchyma layers are evident, and five extra vascular bundles—each associated with a stamen—are observed. At this level, the stamens are adnate to the corolla. The solid style is composed of an epidermal layer enclosing parenchymatic cells, a central transmitting tissue, and two vascular bundles. In sections above the ovary, numerous pappus elements and the central transmitting tissue are visible (Figure 3h).

**Figure 4:**
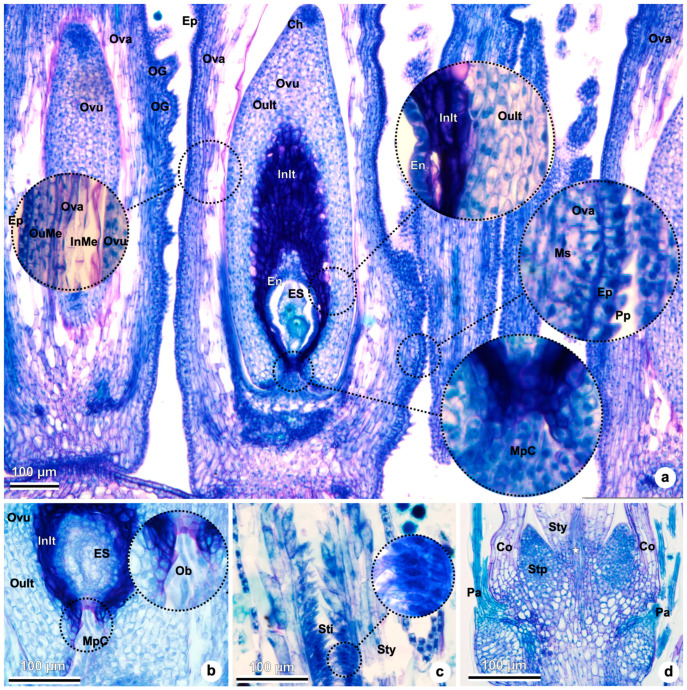
Gynoecium anatomy of *Taraxacum kok-saghyz* observed in longitudinal views in light microscopy. (**a**) Ovaries showing the outer epidermis with conical papillae. Outgrowths on the surface of the ovary. Ovary outer and inner mesophylls with differentiations. Micropylar canal and chalaza. Outer and inner zones of the integument of the ovule and endothelium. Embryo-sac showing the central and the egg cell. (**b**) Ovary outer and inner zones of the integument. In the detail, the micropylar canal showing an obturator. (**c**) Style with two stylar branches and stigmatic tissue with papillate cells. (**d**) Stylopodium at the base of the style, the base of the corolla, and the insertion of the pappus. Pollen tube transmitting tissue (white star). Abbreviations: CC, central cell; Ch, chalaza; Co, corolla; EC, egg cell; En, endothelium; Ep, epidermis; ES, embryo-sac; InIt, inner zone of the integument; InMe, inner mesophyll; OG, outgrowths; MpC, micropylar canal; Ms, mesophyll; Ob, obturator; OuIt, outer zone of the integument; OuMe, outer mesophyll; Ova, ovary; Ovu, ovule; Pa, pappus; Pp, papillae; Sti, stigma; Stp, stylopodium; Sty, style.

The gynoecium is bicarpellary and syncarpic. The ovary is inferior (Figure 3a–c,i), unilocular, uniovulate, and with basal placentation (Figure 4a). Its surface shows many outgrowths, a single-layered outer epidermis, and a disintegrated inner epidermis (Figure 3i and Figure 4a).

Early in development, ovary mesophyll is undifferentiated; only the epidermal layers are recognized. Later, the layers of the ovary mesophyll differentiate (Figure 3i and Figure 4a). The outer layers of the ovary mesophyll are formed by smaller cells and more compactly arranged parenchymatic cells with denser cytoplasm. In contrast, the inner layers are composed of spongy parenchyma, composed of more elongated cells (Figure 3i and Figure 4a). Outer epidermal layer epidermal layer cells in the ovary with conical papillae (Figure 4a).

The ovule is anatropous (Figure 3c and Figure 4a,b) and shows a chalazal region characterized by a group of smaller cells (Figure 4a) and the micropylar end in the opposite direction (Figure 4a,b). The rapheal bundle is observed in the raphe and anti-raphe regions of the ovule (Figure 3i).

The integument of the ovule is formed by distinct zones of cells (Figure 3i and Figure 4a,b). The inner zone of the integument (also known as the periendothelial zone) show cells with thicker walls and denser cytoplasm than the outer zone. The inner epidermal cells of the integument form an endothelium layer (also known as the integumentary tapetum) that surrounds the embryo sac (Figure 3i and Figure 4a,b). The micropylar canal is detailed in Figure 4b, with the elongated cells of the micropylar obturator also visible.

The style is filiform and distally divides into two stylar branches. The stigmatic tissue, densely covered with papillae, and the pollen tube transmitting tissue are clearly visible at this level (Figure 4c,d). At the base of the style, the stylopodium is present, along with the base of the corolla and the point of pappus insertion (Figure 4d).

### 2.3. Anther Wall Formation, Microsporogenesis, and Microgametogenesis

**Figure 5:**
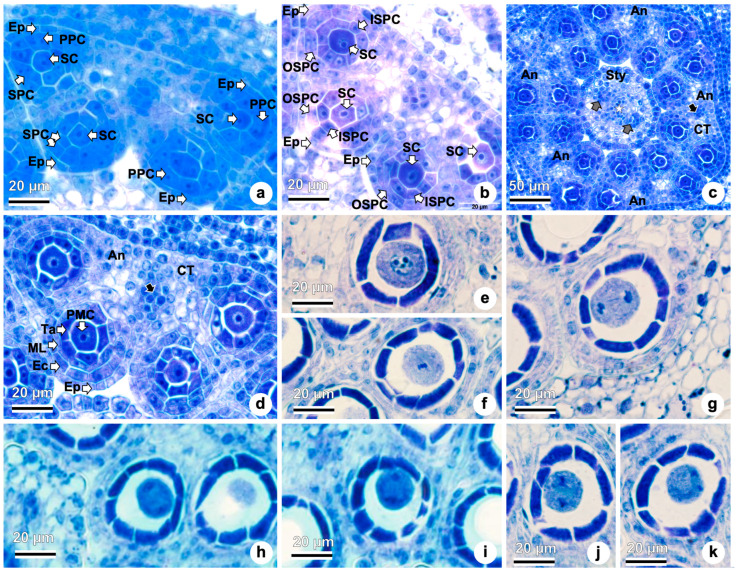
Anther wall formation and microsporogenesis of *Taraxacum kok-saghyz*. (**a**–**k**) Transverse views in light microscopy of anthers. (**a**) Anther wall is surrounded by epidermal cells, primary parietal cells, primary sporogenous cells, secondary parietal cells. (**b**) Outer secondary parietal cells, inner secondary parietal cells (**c**) Five stamens with anthers differentiated into two thecae, each with two pollen sacs, showing the connective tissue and the filament (black arrow). Style is with two lateral vascular bundles (gray arrow) and the pollen tube transmitting tissue (star). (**d**) Magnified view of the Figure 5c. Anther with epidermis, endothecium, middle layer, tapetum, and pollen mother cells. (**e**) Prophase I. (**f**) Metaphase I. (**g**) Late anaphase I. (**h**) Telophase I. (**i**) Prophase II. (**j**) Metaphase II. (**k**) Late anaphase II. Abbreviations: An, anthers; CT, connective tissue; Ec, endothecium; Ep, epidermis; ISPC, inner secondary parietal cell; PMC, pollen mother cell; ML, middle layer; OSPC, outer secondary parietal cell; PPC, primary parietal cell, SC, sporogenous cells; SPC, secondary parietal cell; Sty, style; Ta, tapetum. White arrows indicate the individual cells/structures labeled on the side.

Each flower has five stamens, and each stamen has an anther differentiated into two thecae, each with two pollen sacs. The anther wall is surrounded by epidermal cells (Figure 5a). Connective tissue connects each theca to the filament (Figure 5a,b). Surrounded by the anthers, the style is observed, with two lateral vascular bundles and the pollen tube transmitting tissue in the middle (Figure 5a,c).

**Figure 6:**
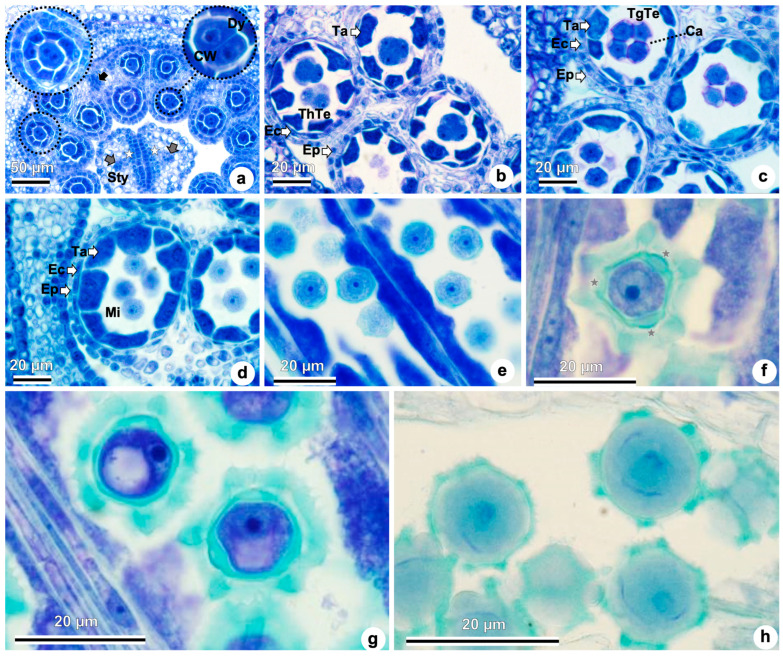
Microsporogenesis and microgametogenesis of *Taraxacum kok-saghyz*. (**a**–**h**) Light microscopy. (**a**) Anthers differentiated into two thecae, each with two pollen sacs, showing the connective tissue and the filament (black arrow), showing simultaneous microsporogenesis (detail on the left), i.e., cell plates/walls form only after meiosis II and a transient four-nucleate stage occurs, and successive microsporogenesis (detail on the right), i.e., cytokinesis occurs after meiosis I–forming dyads, and again after meiosis II. Right: dyad separated by a complete cell wall. The style shows two lateral vascular bundles (gray arrow) and the pollen tube transmitting tissue (asterisk). (**b**) Tetrahedral microspore tetrads; disintegration of the middle layer. (**c**) Tetragonal microspore tetrads joined by callose. (**d**) Free microspores. (**e**) Free microspores with starch accumulation. (**f**) Free microspore prior to mitosis showing three apertures (asterisks). (**g**) Vacuolated microspore with the nucleus displaced toward the cell wall (parietal nucleus). Tapetal cells degenerated; endothecium lacking secondary wall thickenings. (**h**) Mature pollen grains are tricellular, consisting of one vegetative cell and two sperm cells. Abbreviations: Ca, callose; CW, cell wall; Dy, dyad; Ec, endothecium; Ep, epidermis; Mi, microspore; Sty, style; Ta, tapetum; TgTe, tetragonal tetrad; ThTe, tetrahedral tetrad. White arrows indicate the individual cells/structures labeled on the side.

Beneath the epidermis, a single row of archesporial cells differentiate in the microsporangium and divide periclinally, forming the primary parietal cells externally and the primary sporogenous cells internally (Figure 5a). The primary parietal cells divide periclinally and anticlinally, forming two layers of secondary parietal cells (Figure 5a,b). The outer secondary parietal cells (Figure 5b) develop into the endothecium and the middle layer (Figure 5c,d). The inner secondary parietal cells (Figure 5b) develop into the tapetum (Figure 5c,d), and the anther wall layers are composed of epidermis, endothecium, middle layer, and tapetum (Figure 5c,d). The epidermis is a single-layered external covering of the sporangial wall (Figure 5a–d). At this stage, the tapetum is formed by uninuclear cells (Figure 5c,d). The middle layers will eventually crush and degenerate during pollen development (Figure 6c,d).

The diploid pollen mother cell (microsporocyte) undergoes meiosis, and the following stages were observed: prophase I (Figure 5e), metaphase I (Figure 5f), late anaphase I (Figure 5g), telophase I (Figure 5h), prophase II (Figure 5i), metaphase II (Figure 5j), and late anaphase II (Figure 5k). Following the completion of meiosis I and II, microspores are organized into tetrads.

Following meiosis I, some pollen mother cells showed cell wall formation, indicating cytokinesis and suggesting a successive type of microsporogenesis, which leads to the dyad stage (Figure 6a). Such dyads, although not systematically quantified, were rarely observed. Notwithstanding, in most dividing pollen mother cells, a cell wall did not separate the two nuclei—feature consistent with simultaneous-type microsporogenesis (Figure 6a). Tetrahedral tetrads were observed during later stages (Figure 6b), at which point the middle layer began to disintegrate, leaving only the epidermis, endothecium, and tapetum. Tetragonal tetrads of microspores were also occasionally detected (Figure 6c).

Tetrads of microspores remain enclosed in callose walls (Figure 6c) until the callose is enzymatically degraded, allowing the release of individual microspores (Figure 6d). At this stage, tapetal cells are already multinucleate (Figure 6b,d). Free microspores begin to accumulate starch prior to mitosis (Figure 6e). Although the study was not designed for this purpose, incidentally, in some sections, some microspores, allowed the visualization of three distinct apertures (Figure 6f), characteristic of tricolporate pollen, which does not exclude the possibility that other types may occur at lower frequencies, given the variation in cytokinesis type and tetrad shape. In Figure 6g, vacuolated microspores are visible, with the nucleus displaced toward the periphery—suggesting the onset of polarity in the developing pollen grain. At this stage, tapetal cells appear degenerated, and the endothecium lacks visible secondary wall thickenings. As development proceeds, each microspore undergoes asymmetric mitosis, producing a larger vegetative cell and a smaller generative cell. The generative cell will later divide to form two sperm cells. Mature pollen grains are tricellular, composed of one vegetative cell and two sperm cells surrounded by a well-developed exine (Figure 6h).

### 2.4. Megasporogenesis and Megagametogenesis

**Figure 7:**
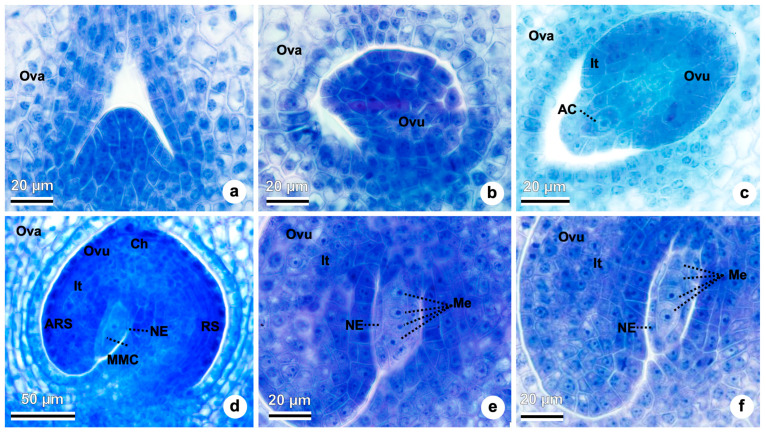
Megasporogenesis in *Taraxacum kok-saghyz*. (**a**) Initial formation of the single ovule. (**b**) Ovule starts curving. (**c**) Ovule integument formation and archesporial cell. (**d**) Megaspore mother cell surrounded by one layer of nucellar epidermis. Chalazal region, integuments, rapheal and antirapheal sides of the ovule observed. (**e**) Tetrad of megaspores formed. (**f**) Tetrad of megaspores formed. Abbreviations: AC, archesporial cell; ARS; antirapheal side; Ch, chalaza; It, integument; Me, megaspore; MMC, megaspore mother cell; NE, nucellar epidermis; Ova, ovary; Ovu, ovule; RS, rapheal side.

Inside the ovary, a single ovule is formed (Figure 7a) with basal placenta. The ovule then curves and reshapes into a protrusion of cells, forming the integument (Figure 7b,c). The ovules are unitegmic and tenuinucellar. In Figure 7c, the formation of the integument is observed. Only one cell within the subepidermal layer of the nucellus differentiates, with a distinct nucleus and dense cytoplasm, constituting the sole archesporial cell (Figure 7c) that later forms the megaspore mother cell (Figure 7d). The megaspore mother cell is recognized by its greater size. It is surrounded by one layer of nucellar epidermis (Figure 7d). The integument overgrows the nucellus (Figure 7d). The rapheal side of the ovule is on the right side and the antirapheal side is on the left side (Figure 7d). The diploid megaspore mother cell soon elongates (Figure 7d), undergoes meiosis, and originates a linear tetrad of haploid megaspores (Figure 7e). The anatropous ovule is already curved to 180º (Figure 7e,f) compared to the initial position (Figure 7a). The internal zone of the integument (the inner epidermal cells) is in contact with the nucellar epidermis and will further differentiate into elongated tangentially endothelium cells with dense cytoplasm (Figure 7e,f). The obturator with elongated cells can be seen in the micropylar region (Figure 7e,f). Typically, following megasporogenesis, three of the four megaspores degenerate, leaving only one functional. In *Taraxacum kok-saghyz*, the chalazal megaspore is most often the functional one, as observed in Figure 7e, where the micropylar megaspores appear to be undergoing degeneration. However, Figure 7f shows a less common configuration, in which the micropylar megaspore remains functional while the others degenerate.

**Figure 8:**
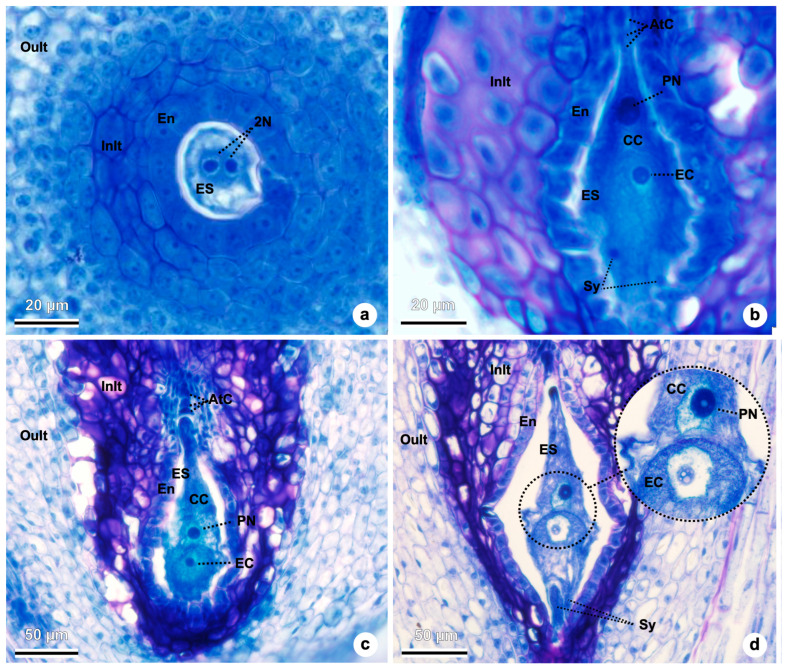
Megagametogenesis in *Taraxacum kok-saghyz*. (**a**) Ovule in transversal view, showing a 2-nucleated embryo sac. (**b**) Embryo-sac is observed with three antipodal cells, the central cell, the egg cell, and two synergids. (**c**) Embryo-sac with the egg cell, the central cell (with one polar nucleus visible), and three antipodal cells at the chalazal pole are observed. (**d**) Mature embryo sac with two synergids in the micropylar end of the ovule, the egg cell, and the central cell (with one polar nucleus visible). Abbreviations: 2N, 2-nucleated embryo sac; AtC, antipodal cells; CC, central cell; EC, egg cell; En, endothelium; ES, embryo-sac; InIt, inner zone of the integument; OuIt, outer zone of the integument; PN, polar nuclei; Sy, synergids.

The functional megaspore undergoes three rounds of mitotic division, producing a 2-, 4-, and eventually an 8-nucleated embryo sac (Figure 8a–d), which subsequently undergoes cellularization (Figure 8b–d). In the transverse section shown in Figure 8a, 2-nucleated stage is visible. Figure 8b shows a nearly complete embryo sac with three antipodal cells at the chalazal end, a large central cell containing the polar nuclei, the egg cell, and two synergids at the micropylar end. At the eight-nucleate stage, but before cellularization (Figure 8b), the nucellar epidermis shows visible signs of degeneration in our sections, becoming more evident in subsequent stages (Figure 8c,d). In Figure 8c, the egg cell, central cell (with one polar nucleus visible), and three antipodal cells are identifiable, although the synergids are not seen in this focal plane. Figure 8d displays a mature, cellularized embryo sac with two synergids at the micropylar pole, an egg cell, and a central cell containing one visible polar nucleus.

## 3. Discussion

Our results broadly support the hypothesis that TK’s reproductive development is consistent with diploid, sexually reproducing *Taraxacum* species and other Asteraceae, providing a histological framework that distinguishes TK from apomictic taxa. At the same time, two notable deviations—occasional successive microsporogenesis and a minor frequency of functional micropylar megaspores—reveal reproductive plasticity that may be relevant to TK’s breeding and population biology.

In our anatomical observations of the inflorescence in TK, we identified six vascular bundles in the corolla, a feature typical of many Cichorieae species [12]. In this group, the corolla tube generally contains five veins, with one bifurcating to form a total of six. Two veins occur near the margins, while the remaining four are positioned between the five corolla teeth.

Surrounding the corolla, we observed the pappus, modified calyx structures that function in herbivore defense and achene dispersal [26]. In TK, we observed homogamous and elongated pappus elements, forming a ring inserted just below the corolla base and above the ovary, as described in our previous work on floral development [24]. The mature pappus elements measured in this study are consistent with those reported previously in TK [27], where the authors observed sizes ranging from 3.5 to 4.5 mm in length.

We also observed the presence of stylopodia (gynoecial nectaries) located above the ovary in TK flowers. Although not examined in microscopic detail, these structures resemble those described in *Taraxacum belorussicum*, where they appear as cone-shaped formations surrounding the base of the style [15], and are similar to those reported in *Smallanthus sonchifolius* (yacon), another species in the Asteraceae family. The presence of such nectaries is commonly associated with the attraction of pollinators [28]. Nevertheless, a detailed histological study of stylopodia in TK is needed before functional comparisons can be made.

Anther development in TK is marked by introrse dehiscence, a characteristic feature in Asteraceae where the elongating style aids pollen presentation [29]. The tapetum in TK was here identified as glandular, consistent with previous observations in other *Taraxacum* species [30]. In TK, tapetal cells became multinucleated through mitotic divisions without cytokinesis, as observed in *Taraxacum udum* [19]. In both species, tapetal degeneration occurred at the free microspore stage, consistent with typical developmental timing. On the other hand, multinucleated amoeboid tapetal cells are common in Asteraceae, as shown in embryological studies of *Ageratum* [31] and *Aster subulatus* Michx., *Kalimeris indica* (Linn.) Sch.-Bip., *Heteropappus arenarius* Kitamura and *Erigeron annuus* (Linn.) [32].

Microsporogenesis is the process through which a diploid pollen mother cell undergoes meiosis to produce four haploid microspores. In angiosperms, the most common developmental patterns are the simultaneous and successive types, which influence the resulting tetrad shape, such as tetrahedral, tetragonal (isobilateral), linear, decussate, T-shaped, or rhomboidal. However, tetrad form alone is not always predictive of the developmental pattern [33]. Microsporogenesis in TK primarily follows the simultaneous pattern typical of eudicots [33,34], which results in regular tetrahedral tetrads. Occasional successive microsporogenesis was also detected in TK, indicated by callose wall formation after meiosis I and the presence of dyads and tetragonal tetrads. Such variation has been similarly reported in other Asteraceae. For instance, in *Aster subulatus*, *Kalimeris indica*, and *Heteropappus arenarius*, microsporogenesis is typically simultaneous, producing tetrahedral or occasionally decussate tetrads, whereas in *Erigeron annuus*, both simultaneous and successive patterns occur, with dyad formation confirming the latter [32]. Although our microsporogenesis observations were not collected as a quantitative survey, mixed successive/simultaneous cytokinesis is potentially relevant to TK domestication because cytokinesis mode and tetrad geometry can influence pollen aperture patterning, and deviations in male meiosis can produce irregular tetrads (dyads/polyads/unequal microspores) associated with reduced pollen fertility [35]. In *Taraxacum*, irregular tetrads have been reported previously [30], and breeding depends on effective pollen function under a strong self-incompatibility system; thus, any shift in the frequency of abnormal microsporogenesis could reduce crossing success and seed production. Although not formally quantified in the present study, our observations of microsporogenesis variation are aligned with the previous study in *Taraxacum wallichii*, a diploid and sexually reproducing species, which forms more than 90% of normal tetrads [30]. However, pollen viability/germination and aperture-type frequencies were not measured here and should be addressed in targeted follow-up work.

These findings underscore the stability of sexual reproduction in TK, supporting its capacity for functional pollen development and sexual reproduction. Moreover, the observed traits distinguish TK from triploid, apomictic *Taraxacum* species like *T. belorussicum* and *T. udum*, which exhibit highly irregular meiosis and defective microspore development [15,19].

The pollen wall, one of the most complex manifestations of plant cell walls [36], serves a dual function: supporting both plant gametogenesis and fertility. Development of the two main layers is regulated by separate tissues, with the outer exine regulated by the sporophyte (tapetal cells) and the inner intine by the gametophyte [37]. Exine formation is further divided into two stages: the determination of the pollen-wall pattern and the deposition of sporopollenin precursors. The pollen-wall patterning phase depends on the formation of a callosic wall, plasma membrane undulation, and the deposition of primexine [36].

Furthermore, the morphology of the pollen exine in *Taraxacum* may affect the microgametophyte’s fitness for successful dispersal to compatible mates [38]. This aspect is particularly relevant in the case of TK, a sexually reproducing species that is self-incompatible, resulting in an obligate sexual outcrossing strategy [39]. In studies inducing both self- and cross-pollination in TK, the authors observed that fewer pollen grains adhered to the stigma papilla cells in self-pollinated flowers compared to cross-pollinated ones [39]. In addition, the small number of pollen grains that adhered to the stigma papilla cells in the self-pollinated flowers showed limited pollen tube growth and failed to penetrate the stigma. A better understanding of pollen morphology could shed light on the mechanism of self-incompatibility in TK. Several questions remain open for answering, such as how does self-incompatibility works at the tissue level in TK? Do the papillae on the stigma show any structural features that could be related to pollen rejection? Is the transmitting tissue organized in a way consistent with compatible vs. incompatible pollen tube growth?

Recent findings in other *Taraxacum* species, such as *Taraxacum ceratophorum*, indicate that exine traits, particularly pollen spine distance and grain size, are subject to pollinator-mediated sexual selection, which directly influences pollen adherence to the pollinator’s body and its subsequent transport [38]. In sexually reproducing dandelions, these morphological features arise during microgametogenesis and play a critical role in ensuring successful pollen pickup and delivery. Considering that TK is an obligate outcrossing species, it is plausible that similar selective pressures shape its pollen wall architecture, highlighting the developmental and functional relevance of exine morphology in mediating pollen–pollinator and pollen-stigma interactions.

The pollen grain in TK is tricolporate, with three germinal apertures, aligning with descriptions for the eudicot clade. This configuration relates to the evolutionary pattern observed in pollen morphology, where aperture arrangements reflect phylogenetic relationships [36] Studies of Asterales pollen morphology [40] have shown that pollen shape is strongly influenced by phylogeny, with related groups showing similar morphologies. These patterns likely result from short bursts of morphological change during key evolutionary events, rather than gradual evolution. Interestingly, even groups with many species and wide distributions do not necessarily show more pollen diversity [40]. In this view, the aperture pattern in TK likely results more from developmental constraints and evolutionary history than from ecological adaptation.

Although our TK material is diploid and sexual, and we did not assess progeny ploidy, *Taraxacum* as a genus comprises diploid sexuals and apomictic polyploids [41]. Crosses between cytotypes (and residual sexuality in apomicts) can generate offspring with altered ploidy via unreduced gametes [42,43]. Within this broader context, reproductive traits—including pollen characteristics—may vary among cytotypes and developmental conditions. In addition, taxonomic studies in *Taraxacum* have reported within-capitulum heterogeneity in pollen production, with inner florets polliniferous while outer florets may be reduced or apolline in some taxa [44]. This suggests that floret position and developmental timing could influence pollen traits. Because our observations were not stratified by cytotype or by floret position within the capitulum, such effects could not be evaluated here. Future comparative studies explicitly accounting for cytotype and capitulum position would be valuable for informing breeding strategies, agronomic practices, and seed production in TK domestication.

In a study on ovary and ovule anatomy in *Taraxacum linearisquameum* (a diploid sexual species) and *Taraxacum gentile* (a triploid, apomictic species), the authors describe no significant differences in the ovary and ovules between the two species [16]. The anatomical features of the ovule in TK observed in this study are consistent with those described for both sexual and apomictic *Taraxacum* species, characterized by anatropous, unitegmic, and tenuinucellate ovules [16,19].

The ovary mesophyll of TK consists of two distinct regions, in agreement with findings from *T. linearisquameum* (diploid) and *T. gentile* (triploid) [16]. Specifically, the outer mesophyll exhibited fewer intercellular spaces, while the inner mesophyll contained more pronounced intercellular spaces. A similar differentiation of mesophyll regions has also been reported in other Asteraceae species, including *Stifftia chrysantha* J.C.Mikan and *Stifftia fruticosa* (Vell.) D.J.N.Hind & Semir, *Wunderlichia mirabilis* Riedel ex Baker and *Wunderlichia senae* Glaz [45].

TK also display two zones in the integument of the ovule, similar to *T. gentile*, and *T. linearisquameum*, ref. [16] forming the inner and the outer zones of the integument. The cells in the inner zone of the integument in TK are thick-walled, with denser cytoplasm. And the inner epidermal cells of the integument differentiate into elongated endothelium cells with dense cytoplasm, in accordance with previous descriptions in *Taraxacum* species [16]. Similarly, in a study with several sexual and apomictic species [23], the authors report that the periendothelial zone of the integument exhibited cytoplasm-rich cells, and the ultrastructural observations showed plasmodesmata connections during early differentiation. As development progressed, the authors observed the cell walls thickened due to mucilage deposition, and the plasmodesmata were associated with cytoplasmic bridges. These features suggest a nutritive role for these cells in supporting embryo development and may also contribute to the regulation of seed hydration during maturation.

The obturator observed in TK is consistent with previous descriptions in the triploid apomictic *T. belorussicum* [15], where the authors identified it as a group of elongated cells located in the micropylar canal. They also reported that the structure of the micropylar canal was similar in both sexual and apomictic *Taraxacum* species.

A study comparing the micropylar canal in the sexual diploid species *Taraxacum tenuifolium* Koch and *T. linearisquameum* with the apomictic species *Taraxacum officinale* [21], found the structures to be similar. This similarity suggests that the presence of obturator cells may aid in the fertilization of an unreduced egg cell in apomictic species [21].

Furthermore, similar micropylar obturators have been reported in other Asteraceae species, *Stifftia chrysantha* J.C.Mikan, *Stifftia fruticosa* (Vell.) D.J.N.Hind & Semir, *Wunderlichia mirabilis* Riedel ex Baker and *Wunderlichia senae* Glaz. where the cells are radially elongated, with pectic cell walls and phenolic-rich cytoplasm [45].

Although the chalazal megaspore is most often functional in TK, we also observed a rarer configuration in which the micropylar megaspore appeared to become functional (Figure 7f). A precedent exists in the classical embryological study of TK which explicitly reports a rare micropylar functional megaspore [17]. Such deviations may reflect heterochrony in degeneration, local disruption of polarity/cell-wall (including callose) dynamics known to be associated with functional megaspore selection, or stochastic developmental variation.

Finally, in our material, the nucellar epidermis appears to undergo degeneration toward late megagametogenesis, becoming evident from the eight-nucleate, pre-cellularization embryo sac stage. In *Arabidopsis*, pre-fertilization nucellar degeneration is described as a highly ordered, genetically regulated process coordinated with female gametophyte expansion, and has been mapped across defined female-gametophyte stages (e.g., onset around FG3 and progression through FG4) [46]. Whether a comparable regulated program operates in TK remains to be tested and will benefit from targeted staging and marker-based analyses in future work.

## 4. Materials and Methods

### 4.1. Plant Material

Inflorescences of TK in various stages of development were collected, from plants originating from the maternal half-sibling family 305-05 (open-pollinated progeny of a single mother plant, representing the genetic diversity typical of outbred TK populations. They were grown in a greenhouse at the Ohio Agricultural Research and Development Center–The Ohio State University, Wooster, OH, with supplemental lighting, using cones with PRO-MIX^®^ (Premier Growers and Consumers, Quakertown, PA, USA) growing medium during fall. Nine plants were collected at each sampling time, at 30, 33, 36, 39, 42, 45, and 48 days after germination, and a total of 25 inflorescences were analyzed. The peduncles varied from 0 to 16.7 cm long. Plants and inflorescences were dissected using a Leica S6D stereomicroscope (Leica Microsystems, Wetzlar, Germany).

### 4.2. Light Microscopy

Whole inflorescences were fixed in FAA (formalin:acetic acid:ethanol:dH_2_O; 10:5:50:35, *v*/*v*) overnight. Samples were then dehydrated at room temperature in a graded ethanol series: 50% and 70% (15 min each), followed by 85% and 95% (1 h each). Samples were embedded in 2-hydroxyethyl methacrylate (Historesin; Leica Microsystems, Germany), sectioned transversely and longitudinally at 8 μm thickness using a rotary microtome Olympus CUT 4055 (Olympus America Inc., Center Valley, PA, USA), and stained with 0.05% (*w*/*v*) toluidine blue [47]. Stained sections were mounted on slides and photographed using an Olympus BX51 light microscope (Olympus America Inc., Center Valley, PA, USA).

### 4.3. Scanning Electron Microscopy

For scanning electron microscopy (SEM), samples were fixed as described above and dehydrated at room temperature in an ethanol series: 50%, 70%, and 90% (15 min each, once), followed by 100% ethanol (three changes). Samples were then critical-point dried using a Samdri-790 apparatus (Tousimis Research Corporation, Rockville, MD, USA), mounted on aluminum stubs, and sputter-coated with platinum. Inflorescences were examined and imaged using a Hitachi S-3500N scanning electron microscope (Tokyo, Japan)under high vacuum.

## 5. Conclusions

Our observations support the hypothesis that TK’s reproductive development is broadly consistent with sexually reproducing diploid Taraxacum species and other Asteraceae. Microsporogenesis is predominantly simultaneous, producing regular tetrahedral tetrads and leading to tricellular, tricolporate pollen—consistent with its sexual diploid status and functional outcrossing system. Notably, occasional successive microsporogenesis suggests limited developmental variability. Ovule and ovary anatomy, including the presence of an obturator and mesophyll differentiation, align with patterns observed across *Taraxacum* species, regardless of reproductive mode.

Notably, this study reports for the first time in TK: (i) occasional successive microsporogenesis with dyad formation, (ii) the presence of a micropylar obturator, and (iii) confirms previous reports that, in rare cases, the micropylar rather than the chalazal megaspore is functional.

These findings fill critical knowledge gaps in the floral and reproductive biology of TK and provide a robust anatomical framework to support breeding efforts, genetic improvement, and studies on self-incompatibility and pollination. This foundational work enhances our capacity to develop TK as a viable industrial crop and informs broader investigations into reproductive evolution in Asteraceae.

## Data Availability

Data is contained within the article.

## Abbreviations

The following abbreviations are used in this manuscript:

AbEp	abaxial epidermis
AC	archesporial cell
AdEp	adaxial epidermis
An	anthers
ARS	antirapheal side
AtC	antipodal cells
Ca	callose
CC	central cell
Ch	chalaza
Co	corolla
CT	connective tissue
CW	cell wall
Dy	dyad
Ec	endothecium
EC	egg cell
En	endothelium
Ep	epidermis
ES	embryo-sac
Fi	filament
Fl	flowers
IB	involucral bracts
IM	inflorescence meristems
InIt	inner zone of the integument
InMe	inner mesophyll
ISPC	inner secondary parietal cell
It	integument
LP	leaf primordia
MC	meristematic cells
Me	megaspore
MMC	megaspore mother cell
Mi	microspore
ML	middle layer
MpC	micropylar canal
Ms	mesophyll
NE	nucellar epidermis
Ob	obturator
OG	outgrowths
OP	ovule primordia
OuIt	outer zone of the integument
OuMe	outer mesophyll
OSPC	outer secondary parietal cell
Ova	ovary
Ovu	ovule
Pa	pappus
PPC	primary parietal cell
PG	pollen grains
PMC	pollen mother cell
PN	polar nuclei
Pp	papillae
Pq	parenchyma
R	receptacle
RB	rapheal bundle
RS	rapheal side
SC	sporogenous cells
SEM	scanning electron microscopy
SPC	secondary parietal cell
Sta	stamens
Sti	stigma
Stp	stylopodium
Sty	style
Sy	synergids
Ta	tapetum
TgTe	tetragonal tetrad
ThTe	tetrahedral tetrad
TK	*Taraxacum kok-saghyz*
VM	vegetative meristems
2N	2-nucleated embryo sac
